# Personalized Approach Bias Modification Smartphone App (“SWIPE”) to Reduce Alcohol Use Among People Drinking at Hazardous or Harmful Levels: Protocol for an Open-Label Feasibility Study

**DOI:** 10.2196/21278

**Published:** 2020-08-14

**Authors:** Victoria Manning, Hugh Piercy, Joshua Benjamin Bernard Garfield, Dan Ian Lubman

**Affiliations:** 1 Monash Addiction Research Centre Eastern Health Clinical School Monash University Richmond, Victoria Australia; 2 Turning Point Eastern Health Richmond, Victoria Australia

**Keywords:** alcohol, hazardous alcohol use, alcohol use disorder, approach bias modification, cognitive bias modification, smartphone app, eHealth, mobile phone app, mHealth, digital health

## Abstract

**Background:**

Alcohol accounts for 5.1% of the global burden of disease and injury, and approximately 1 in 10 people worldwide develop an alcohol use disorder. Approach bias modification (ABM) is a computerized cognitive training intervention in which patients are trained to “avoid” alcohol-related images and “approach” neutral or positive images. ABM has been shown to reduce alcohol relapse rates when delivered in residential settings (eg, withdrawal management or rehabilitation). However, many people who drink at hazardous or harmful levels do not require residential treatment or choose not to access it (eg, owing to its cost, duration, inconvenience, or concerns about privacy). Smartphone app–delivered ABM could offer a free, convenient intervention to reduce cravings and consumption that is accessible regardless of time and place, and during periods when support is most needed. Importantly, an ABM app could also easily be personalized (eg, allowing participants to select personally relevant images as training stimuli) and gamified (eg, by rewarding participants for the speed and accuracy of responses) to encourage engagement and training completion.

**Objective:**

We aim to test the feasibility and acceptability of “SWIPE,” a gamified, personalized alcohol ABM smartphone app, assess its preliminary effectiveness, and explore in which populations the app shows the strongest indicators of effectiveness.

**Methods:**

We aim to recruit 500 people who drink alcohol at hazardous or harmful levels (Alcohol Use Disorders Identification Test score≥8) and who wish to reduce their drinking. Recruitment will be conducted through social media and websites. The participants’ intended alcohol use goal (reduction or abstinence), motivation to change their consumption, and confidence to change their consumption will be measured prior to training. Participants will be instructed to download the SWIPE app and complete at least 2 ABM sessions per week for 4 weeks. Recruitment and completion rates will be used to assess feasibility. Four weeks after downloading SWIPE, participants will be asked to rate SWIPE’s functionality, esthetics, and quality to assess acceptability. Alcohol consumption, craving, and dependence will be measured prior to commencing the first session of ABM and 4 weeks later to assess whether these variables change significantly over the course of ABM.

**Results:**

We expect to commence recruitment in August 2020 and complete data collection in March 2021.

**Conclusions:**

This will be the first study to test the feasibility, acceptability, and preliminary effectiveness of a personalized, gamified ABM intervention smartphone app for hazardous or harmful drinkers. Results will inform further improvements to the app, as well as the design of a statistically powered randomized controlled trial to test its efficacy relative to a control condition. Ultimately, we hope that SWIPE will extend the benefits of ABM to the millions of individuals who consume alcohol at hazardous levels and wish to reduce their use but cannot or choose not to access treatment.

**Trial Registration:**

Australian New Zealand Clinical Trials Registry (ANZCTR) ACTRN12620000638932p; https://www.anzctr.org.au/Trial/Registration/TrialReview.aspx?ACTRN=12620000638932p

**International Registered Report Identifier (IRRID):**

PRR1-10.2196/21278

## Introduction

Globally, alcohol is estimated to account for 5.1% of the burden of disease and injury, and is associated with health, social, and economic harms that not only impact the drinker but also those around them, including their family, work colleagues, and the broader community [1]. Approximately 1 in 10 adults worldwide have had an alcohol use disorder (AUD) during their lifetime, and it is estimated that 1 in 5 adults in Australia have met the criteria for AUD [2]. Unfortunately, even among those individuals who seek treatment, relapse rates typically range from 55% to 85%, depending on the population being studied, the type of treatment administered, and the definition of relapse used [3-5].

There are numerous factors that may make it difficult for people to reduce or cease their drinking, and recent research suggests that these include learned associations between alcohol and related stimuli, which have a strong influence on behavior at a subconscious level. According to the highly influential incentive-sensitization model [6], repeated use of addictive drugs sensitizes the neural reward system, thereby strengthening the attention-grabbing and motivational properties of drugs and their associated cues [7]. Stimuli associated with drug use (such as physical and social contexts, sights, sounds, and scents) increasingly capture attention (ie, developing an “attentional bias” [8]), resulting in cue-induced cravings [9]. This incentive sensitization process is also purported to lead to the development of “approach bias” (ie, an automatic, impulsive action tendency to approach drug-related cues) [8]. Berridge and Robinson [10] posit that the subconscious aspects of incentive salience may influence behavior in the absence of conscious “wanting,” or even in the presence of a conscious desire to *not* use the drug. Thus, while there is some evidence that alcohol cognitive biases are associated with craving [11], cognitive biases may also influence alcohol consumption even when a drinker does not consciously “want” alcohol. Craving [12,13], approach bias [14], and attention bias [15] have all been found to predict heavy alcohol use and relapse. Since alcohol-related cues are ubiquitous in Western societies, and nearly impossible to avoid, the craving and cognitive biases that can be elicited by these cues pose a serious challenge for people seeking to reduce or cease their drinking.

Research has shown that alcohol approach biases can be reduced, or even reversed, through a form of computerized “brain training” known as approach bias modification (ABM) [16]. ABM works by repeatedly presenting individuals with alcohol-related pictures to which they must make an “avoidance” movement (eg, by pushing away images of alcoholic beverages using a joystick) and nonalcoholic beverage images to which they must make an “approach movement” (by pulling on the joystick). Over time, individuals learn to automatically avoid alcohol-related cues. In one study, completing only six 15-minute ABM training sessions reduced cue-induced neural activity in the amygdala in male patients with AUD, and this reduction in neural activation was associated with reduced self-reported alcohol cravings [17]. Importantly, several randomized controlled trials (RCTs) have shown that when delivered as an adjunctive intervention during residential AUD treatment, 4-6 sessions of ABM can reduce the likelihood of posttreatment relapse [5,16,18,19].

Residential treatment is appropriate for people with severe AUD [20]. However, there is a much larger population of people with less severe alcohol use problems that do not warrant residential treatment, although their alcohol use still poses risks to health and quality of life [21,22]. Thus, expanding the application and availability of ABM beyond the residential settings where its efficacy has been demonstrated could have a more widespread benefit, provided it can be shown to be feasible and effective in these other settings or modes of delivery. One way in which this can be achieved is through the development of smartphone apps, which have several advantages, including the ease of use of smartphones, their wide availability, and the fact that they are already owned by a large number of people [23-25]. By delivering ABM remotely via smartphone, people can complete training sessions at times that are most convenient for them (which could increase the acceptability of ABM) and in any location (where contextual generalization of training effects may be increased by completing ABM in more naturalistic environments rather than in clinical settings). Smartphone ABM would therefore allow people to freely access ABM training, including those who have difficulty accessing, or who are reluctant to access, traditional addiction treatment services.

Thus far, we are aware of only two studies examining ABM smartphone apps, both of which had promising findings. In the United Kingdom, Crane et al [26] tested apps containing various combinations of 5 different modules (including an ABM module) among people drinking at hazardous levels, and found that combinations in which both ABM and normative feedback were included reduced participants’ weekly alcohol consumption. In Germany, Laurens et al [27] piloted an ABM app in people who were concerned about, or wished to reduce, their drinking. Participants were encouraged to complete at least 2 ABM sessions per week over a 3-week period. Although the majority of those who enrolled failed to complete the 3-week posttraining questionnaire, the majority of those who submitted the questionnaire had completed the recommended 6 sessions. Weekly alcohol consumption declined over this 3-week period, and even further at a 3-month follow up (although there was no control group with whom to compare these outcomes, which may have been biased by the high dropout rate). Participants were asked to provide feedback regarding the app, and although the feedback was generally positive, the participants criticized the lack of personalization, as well as the monotony and repetitiveness of the ABM training, suggesting that game-like features could make it more engaging [27].

Participants’ criticism of the lack of personalization of Laurens et al’s [27] app is unsurprising, given that all participants were trained using the same standardized set of beverage images. In our research on AUD treatment-seekers [19,28], we have observed that participants tend to drink a limited range of beverages. Thus, when ABM programs use a standard picture set of beverages for all participants, many images may have little relevance to most individuals (eg, being repeatedly trained to avoid images of beer may have little impact for someone who only drinks wine). Since approach bias is the product of repeated associative conditioning experiences [29], it is likely to be specific to stimuli resembling the drinks frequently consumed by an individual. Designing ABM tasks where individuals can use their own “personalized” images is therefore likely to be more engaging (as previously suggested [27,30]), as well as potentially more “potent” at reducing approach bias. Smartphones can facilitate this personalization by allowing participants to incorporate their own photos of the beverages they most wish to avoid.

In addition to personalizing the “avoidance” stimuli, “approach” stimuli could also be personalized. In almost all alcohol ABM research conducted to date [5,16,18,19,26-28,31], participants have been systematically trained to approach nonalcoholic beverages. These serve as relatively neutral stimuli, well-matched to alcohol-related stimuli in terms of content, and are therefore ideal for laboratory studies examining the psychological mechanisms of ABM. However, they are likely to be monotonous and of relatively little personal relevance to patients [27], and thus may not be ideal for clinical application. Recently, we have begun exploring the use of images representing positive, personal goals (eg, images symbolizing friends, family, social connection, pets, exercise, financial gain) as the “approach” stimuli in ABM training for other substance use disorders [32]. In this way, personalized ABM can simultaneously be used to weaken the motivation to drink while increasing the motivation toward positive goals, which may further increase its overall therapeutic benefit. Indeed, one recent study supports this possibility. In students with a recent history of both risky alcohol use and unprotected casual sex, Hahn et al [33] showed that by training participants to avoid alcohol images and approach condom images, approach bias was both reduced toward alcohol and increased toward condoms. In a smartphone app, people could use their own photographs of friends, family, hobbies, and similar as approach stimuli, making the training task highly tailored to the individual. Including gamified aspects in the task may also improve engagement even further, enhance completion rates, and thereby further enhance efficacy.

We aim to test the feasibility and acceptability of SWIPE, a novel smartphone-delivered personalized ABM app to help reduce alcohol consumption and cravings, in a sample of people reporting hazardous alcohol use (ie, a score of 8 or more on the Alcohol Use Disorders Identification Test (AUDIT), a commonly used AUD screening tool [34]) recruited from the general community. In addition, we aim to gather preliminary data on drinking, alcohol craving, and alcohol dependence severity outcomes following training, and to test whether these outcomes vary according to participants’ demographic and preintervention alcohol use characteristics. This will allow for assessment of whether there are grounds to proceed to an RCT for testing its effectiveness, and identify which population would be best for such an RCT to target.

We have established the following 5 hypotheses. First, we expect to recruit at least 500 participants within 6 months of launching the app, and estimate that at least 60% of participants will complete 8 sessions of ABM, supporting its feasibility. Second, the mean ratings of the app will be greater than 3 on the “functionality,” “esthetics,” and “app subjective quality” subscales of the Mobile Application Rating Scale (MARS) [35], demonstrating adequate acceptability. Third, there will be statistically significant decreases in the number of standard drinks per week, number of days in which alcohol was used in the past 7 days, alcohol craving, and Severity of Dependence Scale (SDS) [36] scores at the end of the 4-week intervention, relative to pretraining scores, suggesting its potential effectiveness. Fourth, there will be dose-response relationships, whereby the degree of reduction between the pretraining and 4-week assessments in measures of alcohol drinking, craving, and dependence severity will be related to the number of ABM sessions completed over this period (ie, more sessions will be associated with larger reductions). Finally, we hypothesize that the reduction of drinking over the intervention period will be larger in those with more severe baseline alcohol use or problems, and will also be larger in those with greater motivation and confidence to reduce alcohol use.

We also intend to explore participants’ reaction time and error rate data from their ABM sessions as this will inform further refinement of the technical parameters of the app after this study is complete.

## Methods

### Design

This is a single-group, open-label feasibility study. Analyses of drinking, craving, and dependence severity will use a repeated-measures design.

### Participants

We aim to recruit a minimum of 500 participants reporting hazardous alcohol use through social media and other online advertising. Participants must be aged 18 years or older, have an AUDIT score of at least 8, own a recently updated (ie, within the past year) Android or Apple iOS smartphone with an Australian phone number, and wish to reduce their drinking.

### Measures

#### Demographic Information

Participants will be asked to enter their age, gender, and postal code of residence in an online survey hosted on Qualtrics [37].

#### Alcohol Problem Severity

The AUDIT [34] will be used at baseline to measure the severity of alcohol use and related problems during the past year. The SDS [36] will be used to measure the severity of psychological dependence on alcohol in the past month. Since the SDS was initially developed to measure dependence on heroin, cocaine, and amphetamines, the wording of some items will be slightly modified to enhance its relevance to alcohol, similar to the wording used by Gossop et al [38].

#### Motivation and Confidence to Change

The “Readiness Rulers” [39] will be used to measure how important participants feel it is to change their drinking (ie, motivation to change) and how confident they feel in their ability to change. Both motivation and confidence are measured on a 1-10 scale.

#### Alcohol Craving

The frequency scale of the Craving Experience Questionnaire (CEQ) [40] will be used to measure the frequency of alcohol cravings over the past week. This scale consists of 10 items, with each item rated on a scale of 0 (not at all) to 10 (constantly). This scale can further be broken down into 3 factors: “intensity,” “imagery,” and “intrusiveness" [40].

In addition to the CEQ, we will also utilize a single-item visual analog scale (VAS) to measure the current intensity of alcohol craving immediately before and after each ABM session. Participants will be asked “How strongly are you craving alcohol right now?” with a line displayed below the question and a slider that they can place between ends anchored with the words “not at all” on the left end and “extremely” on the right. A participant’s placement of the slider will be converted to a number ranging from 0 to 100.

#### Alcohol Consumption

At baseline, participants will be asked to estimate the number of days on which they consumed alcohol out of the past 28 days. In addition, they will be asked to use a calendar chart to enter the number of standard drinks consumed on each of the past 7 days so that the total amount of alcohol consumed in the past week can be calculated. To improve accuracy of the self-report, an infographic showing how much wine, beer, or spirits corresponds to one standard drink will be displayed with the calendar chart, and this infographic will also contain a link to the Australian Government’s Department of Health standard drinks guide [41]. This 7-day drinking assessment will be repeated at weekly intervals over the course of the intervention. Twenty-eight days after completing the intervention, participants will again be required to complete the alcohol consumption calendar chart, where they will estimate the number of days on which they consumed alcohol out of the past 28 days, and the number of standard drinks consumed on each day in the past week.

#### App Acceptability

At the end of the 4-week intervention, participants will be asked to complete the “functionality,” “esthetics,” and “app subjective quality” subscales of the user version of the MARS (uMARS) [35]. Additionally, participants have the option to enter free text in response to 3 open-ended questions: “What did you like about this app?” “What did you not like about the app?” and “Any further comments about the app?”

### Intervention

Prior to commencing the intervention, participants will be prompted to select 6 alcohol-related pictures that represent the drinks they most frequently consume. Participants can either take photographs using their phone or select pictures from a library of 72 alcohol-related images chosen to represent a broad range of alcoholic beverages and brands commonly consumed in Australia. Participants will then be prompted to select 6 pictures that “represent your goals and motivations.” Again, participants can either use photographs from their phone or select pictures from a library of 72 pictures representing a range of healthy activities or positive goals and sources of pleasure (including family or friends enjoying time together; financial success; employment; exercise, sports, and recreational activities; healthy foods; pets; travel and holidays) that do not contain any depiction of alcohol. Images included in the alcohol and positive image libraries were selected in consultation with a focus group of people (N=5) with lived experience of treatment for alcohol use problems (see further details below). It should be noted that if participants use their own photographs, these images are not uploaded to a SWIPE server. To maintain their privacy, images remain stored only on the participant’s phone, and the SWIPE app only uses these files locally while the participant is completing a training session.

Once the participant selects their 12 pictures, they will be presented with instructions for the ABM task. Pictures will be displayed with a white frame around them, which will be in either landscape or portrait orientation. When the frame is in landscape orientation, the participant is required to swipe downward (ie, toward themselves), which causes the picture to expand, as if the participant has pulled the picture toward themselves. When the frame is in portrait orientation, the participant is instructed to swipe upward (ie, away from themselves), which causes the picture to shrink until it disappears, as if they have pushed it away. If the participant swipes in the wrong direction, a red “X” is displayed to inform them that they made an error. Additional technical details regarding image display (including image size, swipe movement criterion, rate of image size change after a swipe response, and interstimulus interval) are reported in the Australian New Zealand Clinical Trials Registry [42].

Following the display of the instructions, participants complete 10 practice trials (including 5 images in portrait frames and 5 in landscape frames, in random order) to familiarize them with the task before commencing the first session of ABM. Each session consists of 156 trials, comprising 13 presentations of each picture. For alcohol pictures, 12 of the 13 presentations are framed in portrait orientation and one is framed in landscape orientation. This is reversed for positive pictures, whereby 12 of the 13 presentations of each positive picture are framed in landscape orientation and one is framed in portrait orientation. Thus, participants should push away 92.3% of alcohol images and pull 92.3% of positive images toward themselves. If participants make the incorrect movement, they are informed that it was an error, but the trial is not repeated.

To increase engagement and encourage participants to respond both quickly and accurately, the task is gamified with a scoring system. Each time the participant swipes an image in the correct direction, they are awarded 10 points. Additionally, they score bonus points for correct responses if their response is fast enough. They will receive 30 bonus points (yielding a total of 40 points for that trial) If they swipe correctly and within 500 milliseconds of picture onset, 20 bonus points (ie, 30 points total) if they swipe correctly within 500-1000 milliseconds, and 10 bonus points (ie, 20 points total) if they respond correctly within 1000-1500 milliseconds. Correct responses that are slower than 1500 milliseconds following picture onset earn only 10 points. If they swipe an image incorrectly (ie, swipe down for portrait or swipe up for landscape), they lose 100 points, regardless of their reaction time.

Participants’ scores will be displayed on the screen as they perform the task. Upon completion of the task, the final score is displayed. On the second and subsequent sessions, participants’ previous session score and the score of their highest-scoring session will be displayed after completing the task so they can compare their performance to previous sessions.

### Consumer Input

Prior to finalizing the app for this trial, we conducted two rounds of consumer consultation with different groups of people who have lived experience of alcohol use problems. The first was a focus group of people with a lived experience of AUD treatment (N=5). This group assisted us in finalizing the alcohol and positive image libraries by reviewing images we were considering including, providing feedback regarding their relevance and appropriateness, and suggesting other imagery to include. This focus group study was approved by the Monash University Human Research Ethics Committee (MUHREC; project 23287). When an initial version of the app was then developed, we pilot tested it with a group of people (N=7) who identified themselves as having unsuccessfully tried to control or reduce their drinking, who were recruited from the general community via social media advertising. We conducted one-on-one interviews with these participants, seeking feedback regarding the functionality and acceptability of the app. Several changes were then made in response to their feedback to make the user interface more convenient and to improve the app’s wording. This pilot testing was approved by the MUHREC (project 23022).

### Procedure

Individuals interested in participating in the study will be directed by social media and online advertising to an online survey hosted by Qualtrics. Participant information will be displayed along with the option to provide consent to participate. The intended schedule of assessments and intervention for those who provide consent is shown in Table 1. Those who agree to participate then proceed to a survey that will screen for eligibility and collect information regarding alcohol problem severity and craving (ie, demographic questionnaire, a question asking them to confirm that they wish to reduce or cease drinking, AUDIT, Readiness Rulers, SDS, and CEQ). If a participant’s total score on the “dependence” items of the AUDIT (ie, items 4, 5, and 6) is at least 4, contact details for a national addiction telephone helpline service will be displayed. Those screened as eligible will be required to provide their mobile phone number in order to receive a link via SMS text message to download the SWIPE app from the Apple or Google Play Store. Upon first opening SWIPE, they will be prompted to provide information about their past-month and past-week alcohol use. Participants are then prompted to upload or select their alcohol-related and positive pictures and will proceed to the first session of ABM. Each session of ABM is immediately preceded and followed by a VAS craving rating. If a participant’s postsession VAS score is 90 or above after any session, contact details for a national addiction helpline service will be displayed.

Participants will be prompted by app notifications to complete a minimum of two ABM sessions each week for 4 weeks. In addition, every 7 days, participants will be prompted to report the number of standard drinks consumed on each day of the past week. At the end of the 4-week training protocol, participants will be prompted to complete a second Qualtrics survey, which will include the CEQ, SDS, and uMARS. Participants who complete this posttraining survey will be given the option to provide their contact details to be in a draw to win one of 10 US $70 (AUD 100) gift vouchers. Four weeks after completing training, participants will be prompted to complete a final 1-month follow-up questionnaire that will assess past-month and past-week alcohol consumption. This study has been reviewed and approved by the MUHREC (project number: 21393).

**Table 1:** Overview of SWIPE app study measures and schedule.

Assessment	Study period						
	Baseline	Week 1	Week 2	Week 3	Week 4	Posttest	Follow up
Demographics	X						
AUDIT^a^	X						
Readiness Rulers	X						
SDS^b^	X					X	
CEQ^c^	X					X	
uMARS^d^						X	
28-day TLFB^e^ (drinking days)	X						X
7-day TLFB (standard drinks)	X	X	X	X	X		X
ABM^f^ intervention		X X	X X	X X	X X		

^a^AUDIT: Alcohol Use Disorders Identification Test.

^b^SDS: Severity of Dependence Scale.

^c^CEQ: Craving Experience Questionnaire.

^d^uMARS: User version of the Mobile Acceptability Rating Scale.

^e^TLFB: timeline followback.

^f^ABM: approach bias modification.

### Primary Outcomes

The primary outcomes will be the number of sessions completed, the proportion of participants who complete 8 sessions of ABM, and the number of days of alcohol use in the past 7 days. The primary time point for all 3 of these outcomes is 4 weeks after a participant commences using the app. Secondary time points for past-week alcohol use will be 1, 2, and 3 weeks since commencing app use and at the 1-month follow up.

### Secondary Outcomes

uMARS subscale scores will serve as a secondary measure of acceptability, and will be measured at the posttest (ie, 4 weeks after commencing the app). An additional secondary outcome to measure feasibility will be the number of participants recruited within 6 months of launching the app. Secondary outcomes pertaining to alcohol use, dependence, and craving will include: (1) number of days of alcohol use in the past 28 days (primary time point at posttest, secondary time point at the 1-month follow up); (2) total standard drinks consumed in the past 7 days (primary time point at posttest, secondary time points 1, 2, and 3 weeks since commencing the app and at the 1-month follow up); (3) SDS score (primary time point at posttest); (4) CEQ score (primary time point at posttest); (5) craving VAS score (primary time point immediately after the final session of ABM).

Additional secondary outcomes will include trial error rates, reaction times, and session durations over the course of all ABM sessions.

### Data Management

Demographic, AUDIT, Readiness Rulers, SDS, CEQ, and uMARS data will be stored in a password-protected online Qualtrics database, from where it will be downloaded for storage and analysis on a password-protected shared drive controlled by Turning Point, an addiction treatment, research, and workforce training organization run by Eastern Health (a public health service in Melbourne, Australia). At posttest, participants will be asked to provide the mobile phone number used to sign up to the app to allow for matching of pretest and posttest responses at the individual level. Alcohol use data and backend user metrics (number of sessions commenced, number completed, session duration, session total score, trial reaction time, and error data) will be stored on a secure Google Firebase server, which will be downloaded for storage and analysis on a password-protected shared drive controlled by Turning Point at the end of the study.

### Statistical Analysis

Feasibility and acceptability will be assessed using descriptive data, including the number of participants recruited, number of sessions commenced, number of sessions completed, and means and distributions of uMARS scores (for each uMARS subscale). Changes in alcohol consumption, craving, and SDS scores will be analyzed using linear mixed modeling. To analyze whether there is a dose-response relationship between the number of ABM sessions completed and these outcomes, we will examine a model including the interaction term between number of sessions and time, which tests whether the number of sessions moderates the effect of time on these outcomes. Similarly, we will examine models containing interaction terms between time and other potential moderators of interest (baseline AUDIT, SDS, CEQ, or Readiness Ruler scores; baseline alcohol use days or standard drinks; whether participants wanted to completely cease vs only reduce alcohol use; demographic variables) to examine whether changes in alcohol consumption, craving, or dependence severity are dependent on any of these factors. To inform refinement of task and scoring parameters for future versions of the app, we will examine rates of errors and distributions of reaction times for each image type. We will also examine the mean and distribution of session durations.

### Statistical Power for Analyses of Alcohol Outcomes

We are likely to have very high power to detect main effects of time on alcohol-related outcomes. A similar study by Laurens et al [27] found that that participants’ weekly consumption of alcohol declined by 0.36 standard deviations postintervention relative to their baseline levels (baseline mean standard drinks per week 33.3, SD 21.8, and mean decline of 7.8 standard drinks per week at posttest). Changes of approximately this effect size (ie, 0.3-0.4) on alcohol-related measures (eg, days of use, number of standard drinks, SDS, CEQ) would be of modest clinical significance. A sample of only 119 would provide 90% power to detect changes of this magnitude using α=.05. We anticipate that at least 300 (ie, 60% of 500) participants will complete the posttest, but even if we achieve the much lower posttest completion rate (37.89%) reported by Laurens et al [27], in a sample of 500, equivalent to 189 participants, we will have 98% power to avoid type 2 errors if the effect size is only 0.3.

It is more difficult to estimate power for the interaction effects we intend to examine in moderation analyses. We conducted 500 simulations, optimistically assuming an overall average effect size of 0.4, but which ranged from 0.1 to 0.7 at the lowest and highest level (respectively) of a uniformly distributed continuous moderator. We also assumed a 0.5 correlation between pre and postintervention values. Under these optimistic assumptions, a posttest sample of 300 would provide approximately 75% power to detect the interaction between the moderator and the effect of time. Thus, moderation analyses are likely to be underpowered if we only recruit 500 participants. Therefore, if we reach our recruitment target early, we will still leave recruitment open until the planned recruitment end date (start of February 2021) to seek a larger sample size for these analyses.

## Results

This project was funded on March 30, 2020 and received approval from the MUHREC on May 29, 2020. As of July 30, 2020, we expect to commence recruitment in mid-August 2020, complete data collection in March 2021, and publish results by the end of 2021.

## Discussion

This study will be a world-first examination of personalized alcohol ABM delivered via a smartphone app. The application of ABM is still in its infancy, despite strong evidence of its efficacy in residential AUD treatment settings [5,16,18,19]. The advantage of smartphone technology is that it allows individuals to engage in neurocognitive training that is designed to dampen impulsive, automatic responding to cues, regardless of time and place. This is the first alcohol ABM study to personalize “avoid” images by using those representing participants’ preferred alcoholic beverages and brands. It is also the first to personalize the “approach” images, following recommendations that these should align with patients’ goals for behavioral change or offer alternative strategies to manage stress (eg, personal health, reconnecting with family and friends, exercise) [30,43-45]. Indeed, this tailored “dual-target” approach (ie, dampening alcohol associations and reinforcing positive motivations) holds promise in light of preliminary evidence that ABM can simultaneously reduce approach bias to an unhealthy behavior (alcohol) and increase approach bias toward a healthy behavior (condom use) in a student sample [33].

There are several practical and logistical issues that may pose potential challenges to successful completion of this study. One limitation is the reliance on self-reported consumption data, including the potential impact of poor recall. We believe the impact of poor recall on reliability of consumption data will be minimized by requiring participants to only recall and report standard drinks in the past week, and that including the standard drink conversion infographic will increase reporting accuracy. Whilst in-person biometric measures to confirm self-reporting are beyond the scope of the current study, we have modeled the assessments closely on the computerized 7-day timeline followback assessment used by Simons et al [46], which showed good concordance with other measures of alcohol use. Nevertheless, some degree of inaccuracy of self-reported data is almost certain despite our measures to minimize it.

It is possible that overall recruitment will be lower than expected, limiting the power of our planned analyses of alcohol use, craving, and dependence outcomes even if completion rates are good. However, we consider this to be unlikely, given that Laurens et al [27] managed to recruit 1082 participants in only 13 days using a similar social media recruitment strategy. However, if recruitment is much slower than expected, we have several additional strategies we can employ. Turning Point, the addiction treatment and research center at which we are based, has a media department that can assist with further promotion of the trial by seeking coverage in other media (eg, radio, newspaper, online news sites). We will also use the professional networks of the authors to enable publicizing the study to more than 3000 alcohol and drug clinicians and other service workers if we need to increase recruitment rates.

Even if the recruitment target is reached, a low rate of completion of postbaseline assessments could still limit our statistical power to analyze alcohol-related outcomes, as well as increase the likely bias of these outcomes. Previous studies of smartphone apps have had very low rates of participants who completed the primary outcome assessment (eg, 27% in Crane et al [26] and 38% in Laurens et al [27]). We hope that offering participants a personalized intervention and incentivizing the posttest questionnaire by offering the opportunity to win a prize will enhance completion rates. Furthermore, to mitigate the risk of poor posttraining assessment completion rates, the app has been designed to include prompts (app notifications) to remind participants to complete assessments. Even if recruitment is faster than anticipated, we will keep recruitment open for the planned 6-month recruitment period to hopefully recruit more than 500 participants, since exploration of potential moderation effects is likely to require larger sample sizes to achieve adequate statistical power.

Nonetheless, even with a majority completing these assessments, outcomes related to craving, alcohol consumption, and app acceptability may be biased by loss of participants; for example, if those who drink more are less likely to complete these measures. We will examine baseline differences between those who complete assessments and those who do not to examine if they differ in terms of alcohol use, AUD severity (ie, AUDIT and SDS scores), and demographic characteristics. However, our main aim in this trial is to test feasibility, and recruitment and completion rates (whether good or poor) will inform assessment of this outcome. Moreover, our findings regarding feasibility, including any problems encountered in this trial, will inform the design of subsequent RCTs aimed at testing the efficacy of this intervention relative to a control condition. Drinking, craving, and dependence outcomes will also inform the design of RCTs by indicating whether certain subpopulations show stronger evidence of effectiveness.

There is a risk that the ABM training, which involves repeatedly responding to alcohol-related images, will have the opposite effect to that intended (ie, triggering cravings [9], potentially leading to increased alcohol consumption). However, in our experience conducting trials of ABM in clinical samples (with much more severe substance use disorder than we anticipate in this trial), rates of withdrawal from participation due to triggering of cravings or distress have been low [19,47]. Moreover, the two previous trials of alcohol ABM smartphone apps found reductions, not increases, in alcohol use, further suggesting that the risk of this unintended, counterproductive outcome is low [26,27]. Nevertheless, as noted above, if participants rate their alcohol craving as very high after a session, the app will display the details of a free 24/7 alcohol and drug counseling telehealth service.

If we find that this intervention is feasible and acceptable, with preliminary evidence of reduced craving or consumption, the findings will inform the design of a large, statistically powered RCT in which its efficacy could be established (eg, with a sham-training or other control condition). This will be a critical next step as its low cost, easy implementation, and wide accessibility means that SWIPE could address the significant gap between the demand for treatment and availability of addiction treatment services [48]. Importantly, this project extends addiction neuroscience-informed interventions beyond the lab and clinic, and translates them into an accessible, easy-to-use tool for the broader community. SWIPE has the potential to deliver a just-in-time intervention during periods of heightened vulnerability (ie, events, days, and times associated with drinking). Although several smartphone apps exist to help individuals reduce their drinking, they predominantly focus on monitoring alcohol consumption and providing normative feedback, while a few also aim to ameliorate psychological processes impaired by heavy alcohol use (eg, long-term planning, decision making) [49]. Because ABM dampens activity in distinct neural pathways that become overactive through heavy alcohol use [17], SWIPE may be a particularly advantageous intervention that is able to benefit heavy drinkers beyond what is afforded by currently available smartphone apps. Additionally, because the training operates by using images of one’s drug of choice (in this case, alcohol), this app could in the future be easily adapted for use with other substances or behaviors that individuals may wish to cut down on (eg, smoking, use of illicit drugs, gambling, gaming).

## Abbreviations

ABM: approach bias modification
AUD: alcohol use disorder
AUDIT: Alcohol Use Disorders Identification Test
CEQ: Craving Experience Questionnaire
MARS: Mobile Application Rating Scale
MUHREC: Monash University Human Research Ethics Committee
SDS: Severity of Dependence Scale
RCT: randomized controlled trial
uMARS: user version of the Mobile Application Rating Scale
VAS: visual analog scale
